# Predicting radiological regression in benign breast hyperplasia: the prolactin-to-estradiol ratio as a prognostic biomarker

**DOI:** 10.3389/fmed.2026.1814991

**Published:** 2026-04-07

**Authors:** Kun Huang, Hongmei Cao, Honglin Du, Lingli Zhang, Huayu Jiang, Feng Yang

**Affiliations:** 1 Department of Thyroid and Breast Surgery, The Second Affiliated Hospital of Guizhou Medical University, Kaili, Guizhou, China; 2 Department of General Medicine, The Second Affiliated Hospital of Guizhou Medical University, Kaili, Guizhou, China

**Keywords:** benign breast hyperplasia, cox regression, hormonal biomarkers, prolactin-to-estradiol ratio, radiological regression, risk stratification

## Abstract

**Background:**

While benign mammary hyperplasia frequently undergoes spontaneous regression, clinicians currently lack validated serological biomarkers for personalized surveillance strategies.

**Objective:**

To evaluate whether the serum prolactin-to-estradiol ratio [PER; prolactin (ng/mL) ÷ estradiol (pg/mL)] can predict radiological regression in benign hyperplasia.

**Methods:**

This retrospective cohort study (January 2020–December 2024) enrolled women (18–55 years) with biopsy-confirmed ductal or lobular hyperplasia. Baseline fasting prolactin and estradiol were measured using duplicate electrochemiluminescence immunoassays (WHO-traceable) from routine clinical samples. Follow-up biopsies were performed only when imaging triggers were met. Multivariable logistic regression and interval-censored Cox models assessed associations with demographic, reproductive, and lesion covariates. Performance was evaluated using C-statistics, calibration, ROC analysis, and decision curves.

**Results:**

Among 1,645 participants completing follow-up (94.7%), 790 (45.5%) demonstrated radiological regression. Patients with regression had significantly lower mean PER compared to non-regressors (0.161 ± 0.086 vs. 0.232 ± 0.136; *p* < 0.001), reflecting a composite hormonal environment of reduced prolactin and relatively elevated estradiol. PER demonstrated a strong inverse dose–response relationship with regression probability (adjusted OR per 0.1-unit increase = 0.15; 95% CI: 0.10–0.22) and time-to-regression (adjusted HR per 0.1-unit decrease = 1.76; 95% CI: 1.54–2.01), with regression rates declining from 60.3% in the lowest PER tertile to 28.0% in the highest. At the optimal cutoff of PER ≤0.185, discriminative performance reached an AUC of 0.664, significantly outperforming clinical variables alone (AUC 0.529), with net clinical benefit confirmed across a broad range of decision thresholds. Predictive effects remained consistent across age, menstrual phase, and histological subtype (all interaction *p* > 0.08), with a modest but significant BMI interaction (*p* = 0.042).

**Conclusion:**

Low PER independently and reliably predicts spontaneous regression of benign breast hyperplasia. External validation studies and point-of-care assay development are needed before clinical implementation.

## 1 Introduction

Usual ductal and lobular hyperplastic lesions of the breast, collectively termed benign mammary hyperplasia, represent the most common non-malignant breast pathology encountered during image-guided biopsies, affecting nearly one-third of women undergoing such procedures. This condition has emerged as the predominant benign breast disorder across East and Southeast Asian populations, where expanding opportunistic screening programs utilizing ultrasonography and mammography have dramatically increased detection rates as part of comprehensive population health initiatives (1, 2). While spontaneous resolution occurs in many hyperplastic lesions, approximately 25% exhibit persistent growth or histological advancement, necessitating repeated imaging studies, multiple biopsies, and causing substantial psychological burden. Critically, longitudinal cohort data demonstrate that non-regressing lesions correlate with a two- to three-fold elevation in subsequent malignancy risk, particularly among lesions displaying proliferative architecture or extensive glandular distribution (3, 4). This creates a challenging clinical scenario: implementing standardized surveillance protocols risks over-monitoring low-risk patients, while surgical intervention in ambiguous cases exposes patients to unnecessary morbidity and healthcare expenditures (5, 6). Therefore, an easily accessible serological biomarker capable of stratifying regression probability would offer immediate clinical value. Current imaging techniques—ultrasonography, mammography, and MRI—detect regression only following macroscopic changes, providing no predictive information. Individual circulating hormones (prolactin or estradiol assessed separately) demonstrate limited and inconsistent prognostic accuracy, highlighting the requirement for a composite metric that reflects the overall endocrine environment (7–11).

Hormonal regulation fundamentally governs benign hyperplasia biology. Estradiol functions via estrogen-receptor-*α* to stimulate ductal epithelial growth and correlates positively with proliferative benign breast pathology and future cancer risk (12, 13). Prolactin, produced by anterior pituitary lactotrophs and local mammary autocrine mechanisms, controls alveolar maturation and cellular survival via PRLR–JAK2–STAT5 pathways and contributes to oncogenesis through enhanced cellular motility, invasiveness, and vascular development (14, 15). Large-scale retrospective epidemiological studies indicate that women with the highest circulating prolactin quartile experience approximately 20–30% increased overall breast cancer risk, with effects most prominent for estrogen-receptor-positive malignancies and magnified among hormone-replacement therapy users (16). Laboratory investigations further reveal intricate crosstalk wherein estradiol upregulates prolactin-receptor expression while prolactin amplifies estrogenic transcriptional activity, suggesting that mammary tissue mitogenic stimulus depends more on relative hormonal equilibrium than absolute concentrations (17, 18).

Despite biological plausibility, evidence supporting the prolactin-to-estradiol ratio (PER) as a prognostic marker remains limited. Earlier small retrospective investigations—predominantly from Western cohorts—have shown inconsistent associations with benign breast conditions including fibrocystic disease and mastalgia, compromised by assay heterogeneity, insufficient menstrual phase control, and single-institution designs (14, 19, 20). Conversely, Asian populations demonstrate distinctive hormonal patterns, including reduced circulating estradiol and elevated prolactin levels, yet PER’s prognostic significance in this demographic remains unexplored (20–22). Furthermore, previous investigations have infrequently incorporated multivariable adjustment, longitudinal modeling, or systematic threshold optimization, thereby restricting clinical utility (23).

China offers a unique and timely setting to investigate hormonal biomarker prognostic value in benign breast pathology (24). Recent demographic transformations—including delayed first pregnancy, reduced fertility rates, and increased hormonal contraceptive usage—have substantially modified endocrine profiles among Chinese women (25–28). Simultaneously, widespread adoption of high-resolution breast ultrasonography and healthcare system modernization have enabled increased biopsy rates for indeterminate findings, generating a substantial, clinically characterized cohort with benign breast hyperplasia (29, 30, 70–72). In this retrospective cohort study, nearly 2,000 Chinese women with histologically confirmed ductal or lobular hyperplasia were enrolled, with pre-biopsy serum samples analyzed for prolactin and estradiol using World Health Organization (WHO) international standard-traceable assays.

This research pursued a hypothesis-driven yet clinically practical approach. Initially, baseline demographic, reproductive, and lesion features were characterized by 12-month regression status, contextualizing hormone distributions against established risk determinants. Subsequently, the independent relationship between PER and regression likelihood was quantified using multivariable logistic and Cox proportional-hazards modeling, supplemented by dose–response analyses exploring linear and threshold relationships. Additionally, diagnostic discrimination, calibration, and net reclassification of PER-based models were compared against conventional clinical models. Finally, subgroup analyses investigated whether PER performance differed across strata defined by age, body composition, menstrual phase, or lesion characteristics, informing potential patient-specific implementation. Essentially, this study aimed to evaluate serum prolactin-to-estradiol ratio prognostic significance in benign breast hyperplasia through comprehensive cross-sectional and longitudinal modeling, rigorous performance validation, and clinically relevant subgroup analysis, ultimately advancing endocrine-guided risk stratification in benign breast disease management.

## 2 Methodology

### 2.1 Study design and participants

The current retrospective cohort study was undertaken at The Second Affiliated Hospital of Guizhou Medical University, Kaili, Guizhou, China from January 2020 through December 2024, following STROBE recommendations (31). Female participants aged 18–55 years with histologically verified breast hyperplasia (conventional ductal or lobular hyperplasia per WHO classifications) were recruited. Participants were excluded if they had prior breast malignancy, high-risk pathology, ongoing hormonal therapy or prolactin-affecting medications, pituitary tumors or hyperprolactinemic disorders, functional ovarian pathology, clinically apparent thyroid disease, adrenal disorders, peri- or postmenopausal status, pregnancy, or lactation within six months.

Eligible participants were identified following independent clinical recommendations for core needle biopsy according to institutional/ACR BI-RADS guidelines (e.g., BI-RADS 4/5, imaging–clinical mismatch, or ≥20% interval enlargement with concerning morphology). Research personnel did not influence biopsy decisions, and no additional tissue cores were obtained for investigational purposes. Power calculations targeted detection of minimum odds ratios of 1.5 (or 0.67 for protective associations) with 80% power and *α* = 0.05, necessitating approximately 1,400 subjects. Our final cohort (*N* = 1,645) provided narrow confidence intervals surrounding the primary effect estimate, demonstrating high statistical precision. Written informed consent specifically addressed research utilization of clinical data and residual specimens plus research blood collection; biopsy determinations and procedures represented standard clinical care and were not study-mandated.

### 2.2 Data collection and laboratory methods

Baseline evaluation encompassed demographics (age, body mass index), reproductive parameters (menstrual phase, parity, active breastfeeding), familial breast cancer history, clinical features (hyperplasia classification and distribution, mastalgia presence, nodule dimensions), and lifestyle variables (smoking habits, alcohol consumption, physical activity levels). No additional blood was drawn for research purposes. Hormonal results (prolactin and estradiol) were extracted from routine clinical specimens obtained during index assessment (standard total volume 3–5 mL per institutional protocols), following overnight fasting between 08:00 and 10:00 h. Laboratory analyses were conducted by the hospital clinical laboratory (Roche Cobas e602, Elecsys®) as standard care; laboratory internal quality control incorporates duplicate measurements/controls within each analytical run. For women with regular cycles, sampling was coordinated to early follicular phase (days 3–7); menstrual phase was documented for all participants, incorporated as a covariate, and assessed for interaction in predetermined stratified analyses. In addition, participants were instructed to avoid supplements, herbal remedies, or medications known to affect prolactin or estradiol levels for 48 h prior to blood sampling.

Hormonal determinations were conducted in duplicate utilizing electrochemiluminescence immunoassays (ECLIA) on Roche Cobas e602 platforms following ICH M10 protocols (32). Prolactin was quantified using Elecsys Prolactin assay (range: 0.047–4,700 ng/mL; inter-assay CV: 2.8%, intra-assay CV: 1.9%). Estradiol was quantified using Elecsys Estradiol III assay (range: 18.4–4,300 pg./mL; inter-assay CV: 3.2%, intra-assay CV: 2.1%). Supplementary hormonal assessments included thyroid-stimulating hormone, luteinizing hormone, follicle-stimulating hormone, progesterone, and testosterone. Metabolic assessments comprised fasting glucose, insulin, total cholesterol, low-density lipoprotein, high-density lipoprotein, triglycerides, vitamin D, and morning/evening cortisol concentrations. Homeostatic model assessment of insulin resistance (HOMA-IR) was computed as fasting glucose × insulin ÷ 405 (33). PER was computed as: serum prolactin (ng/mL) ÷ serum estradiol (pg/mL). This ratio was selected *a priori* because prolactin primarily promotes mitogenesis while estradiol, without strong prolactin signaling, may favor differentiation or apoptosis; the quotient therefore amplifies the biologically meaningful balance while minimizing random intra-cycle variation.

### 2.3 Histological assessment

Baseline histology was derived from standard-of-care, ultrasound-guided 14-gauge core needle biopsy conducted exclusively for clinical indications prior to study enrolment (e.g., imaging suspicion or imaging–clinical discordance); no research-exclusive biopsies were performed. It is critical to note that the baseline tissue core was used solely to establish the entry-level histological diagnosis and was not re-examined for, or in any way used in relation to, follow-up outcome assessment. These two processes were entirely independent: baseline histological classification was derived from the index biopsy specimen, while the primary study outcome (radiological regression at 12 months) was determined exclusively by serial high-resolution ultrasonographic measurements at pre-specified 3-month intervals. Any repeat biopsy undertaken during the follow-up period was performed only when independent imaging-triggered clinical criteria were met—specifically: (i) lesion progression to BI-RADS 4/5; (ii) imaging–clinical discordance; or (iii) maximal diameter increase ≥20% with new or indeterminate morphology—and these specimens constituted entirely new, independent clinical samples distinct from the original diagnostic core. Specimens underwent routine processing and hematoxylin–eosin staining. Two independent breast pathologists (each with >15 years’ experience), masked to clinical and hormonal data, examined all slides; interobserver concordance for hyperplasia classification was excellent (*κ* = 0.91, 95% CI 0.87–0.94). Discrepant interpretations (*n* = 23; 1.4%) were adjudicated by consensus with a third senior pathologist. Hyperplasia was classified according to WHO criteria, and distribution was categorized as focal (<25% of sampled tissue) or diffuse (≥25%) (34). Inter-reader reliability for ultrasonographic assessment of regression was evaluated using a subset of 200 cases reviewed by two independent radiologists, achieving substantial agreement (*κ* = 0.82, 95% CI: 0.76–0.88).

All participants received high-resolution breast ultrasonography every 3 months (±2 weeks). Repeat biopsy was conducted only if (i) the lesion progressed to BI-RADS 4/5, (ii) imaging–clinical discordance existed, or (iii) maximal diameter increased by ≥20% with new or indeterminate morphology. Without these triggers, regression was defined radiologically as complete sonographic disappearance or a ≥ 50% diameter reduction with stable benign characteristics (35–37).

### 2.4 Statistical analysis

All statistical evaluations followed a predetermined analysis plan finalized before database closure. Continuous variables were compared using independent t-tests or Mann–Whitney U tests, and categorical variables using χ^2^ tests or Fisher’s exact tests. The relationship between PER ratio and radiological regression was analyzed using logistic regression models: Model 1 (unadjusted), Model 2 (demographic adjustment), and Model 3 (fully adjusted for all significant covariates). PER ratio was analyzed both continuously (per 0.1-unit change) and categorized into tertiles. Receiver operating characteristic curves were constructed to evaluate discriminative performance, with area under the curve calculated using the DeLong method (38). Optimal thresholds were determined using Youden’s index and other established techniques (39). Predetermined subgroup analyses examined effect modification by age, BMI, menstrual phase, and hyperplasia characteristics using interaction terms. Time-to-regression was modeled with Cox proportional-hazards regression incorporating interval censoring boundaries derived from the ultrasonography schedule. Non-parametric interval-censored survival curves were also estimated using the Turnbull estimator. The proportional-hazards assumption was evaluated by Schoenfeld residuals and log-minus-log plots. Interval censoring was implemented to account for regression timing being observed only within 3-month intervals rather than at precise time points. The interval boundaries were defined as: (0, 3), (3, 6), (6, 9), and (9, 12) months, with participants censored at their final follow-up visit if regression was not observed. The icenReg package in R was used for interval-censored Cox regression analysis.

Internal validity was examined using 1,000 bootstrap resamples with bias-corrected, accelerated confidence intervals, and optimism-adjusted calibration slopes are reported (40). Model performance was assessed using C-statistics, calibration plots, and Hosmer-Lemeshow tests. Complete case analysis was used given <2% missing data, with multiple imputation sensitivity analyses. Multiple testing adjustment was applied to secondary endpoints using the Benjamini-Hochberg method with false discovery rate set at 5%. Primary endpoint analysis (PER association with regression) was not adjusted as it represented the single pre-specified primary hypothesis. Subgroup analyses were considered exploratory and reported with nominal *p*-values alongside adjusted values. Model assumptions were verified through: (1) linearity assessment using restricted cubic splines; (2) independence evaluation through residual analysis; (3) model specification and residual diagnostics (including overdispersion checks); and (4) multicollinearity assessment using variance inflation factors (all VIF < 2.5). Missing data patterns were evaluated using Little’s MCAR test (*p* = 0.342), supporting the missing completely at random assumption. All analyses were performed using R version 4.3.0 with packages: survival (v3.5–5), pROC (v1.18.4), rms (v6.7–0), icenReg (v2.0.15), and mice (v3.16.0) for multiple imputations. Statistical significance was defined as *p* < 0.05 (two-sided).

### 2.5 Quality assurance and ethics

Electronic data capture utilized REDCap with real-time validation checks. Source data verification was conducted for 20% of randomly selected patients. Inter-laboratory standardization was maintained through quarterly proficiency testing. Pathology quality assurance included inter-observer agreement assessment and monthly case reviews. The investigation was conducted according to Good Clinical Practice guidelines with Data and Safety Monitoring Board oversight. Patient confidentiality was maintained through unique study identifiers, and all participants provided written informed consent. All hormone assays underwent rigorous quality control including: (1) daily calibration with certified reference materials; (2) duplicate analysis of 100% of samples with coefficient of variation <5%; (3) quarterly external proficiency testing through the College of American Pathologists; (4) blinded re-analysis of 10% of samples showing <3% inter-assay variation; and (5) temperature monitoring during storage and transport with documented cold-chain maintenance.

## 3 Results

### 3.1 Study population and baseline characteristics

**Figure 1:**
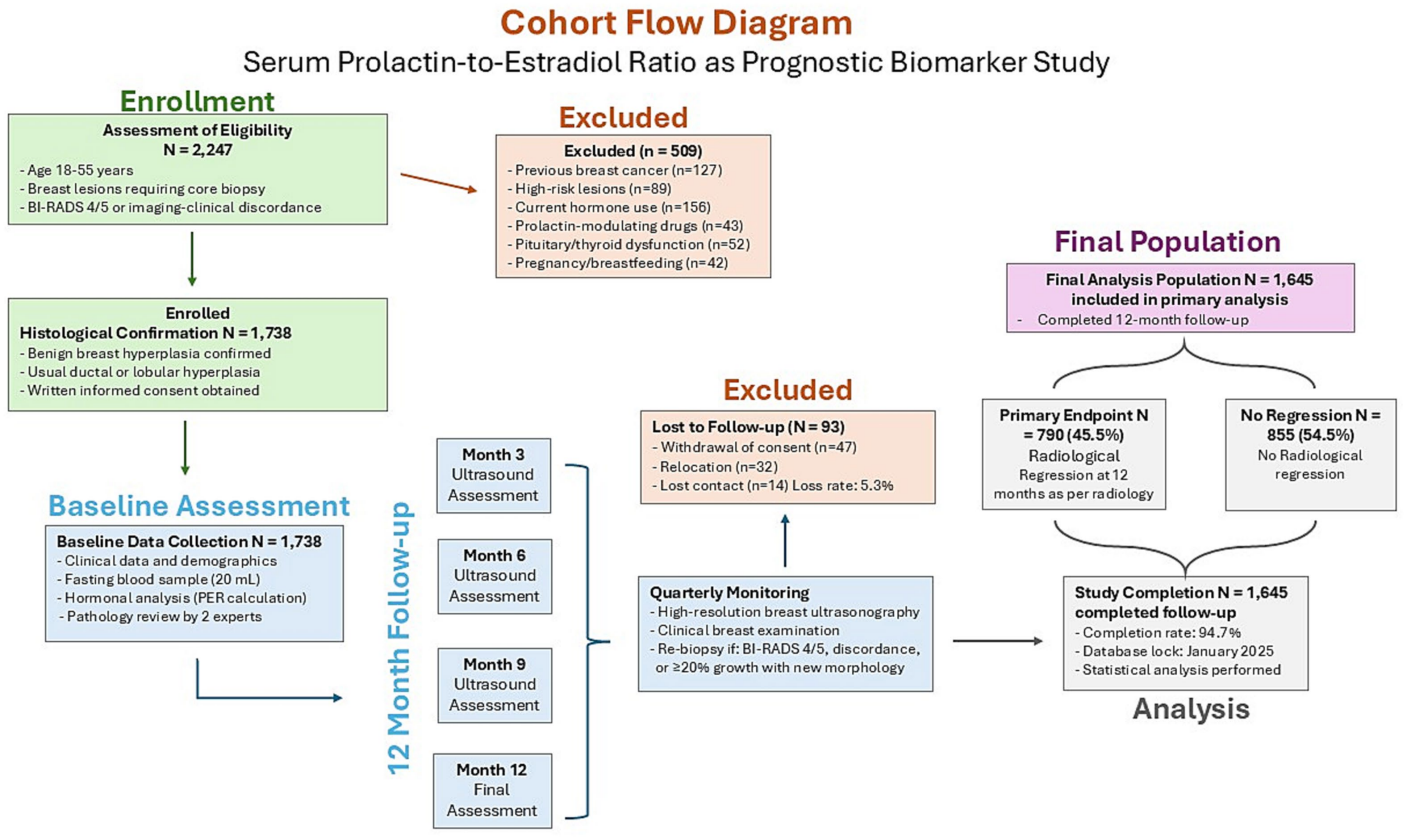
STROBE-compliant retrospective cohort flow for the serum prolactin-to-estradiol ratio (PER) prognostic study.

**Figure 2:**
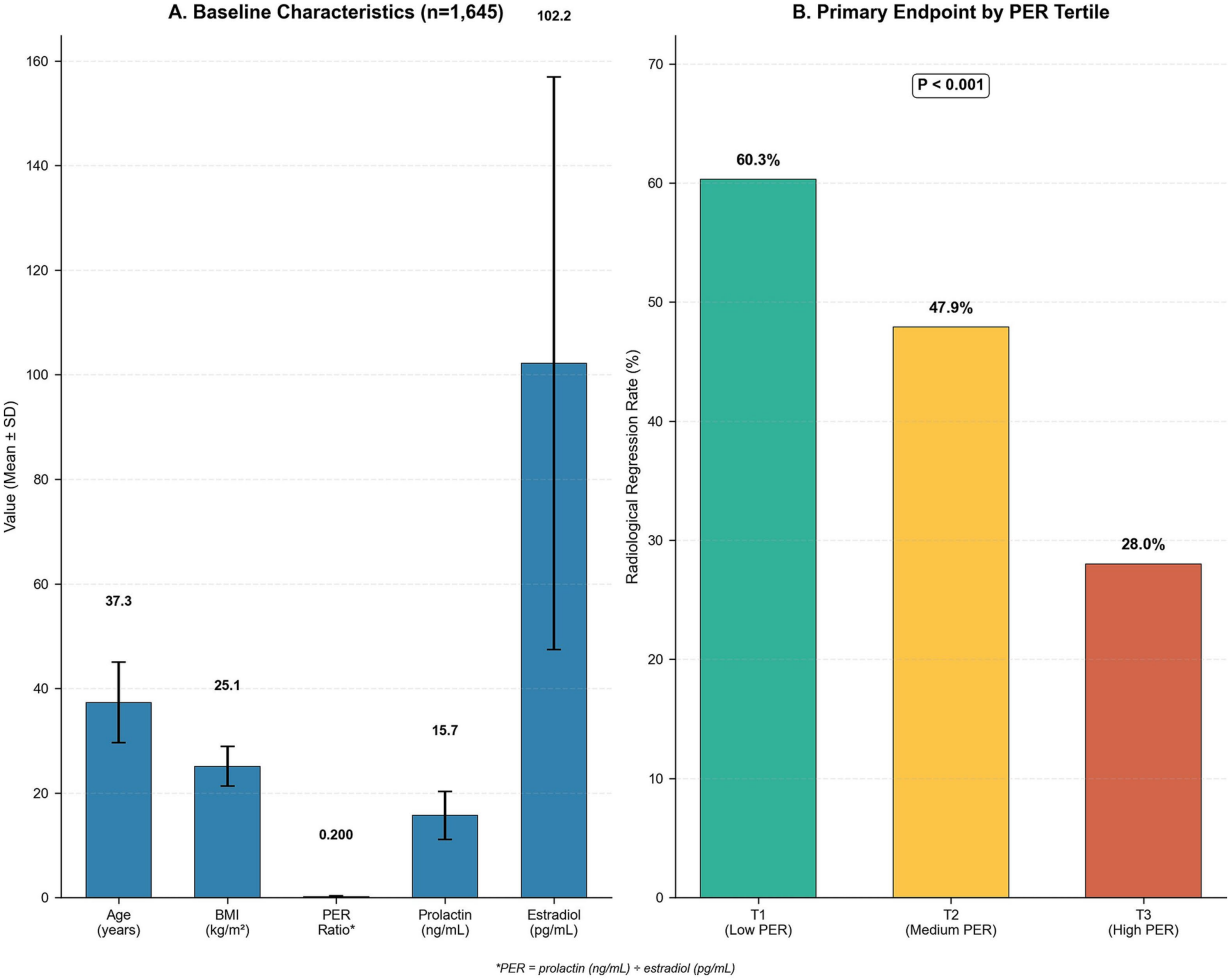
Baseline characteristics and primary endpoint by PER tertile (analysis set *N* = 1,645). **(A)** Bar chart of baseline cohort means (±SD): age 37.3 years, BMI 25.1 kg/m^2^, PER 0.20, prolactin 15.7 ng/mL, estradiol 102.2 pg./mL; error bars indicate SD. **(B)** Twelve-month radiological regression by PER tertile: T1 (≤0.133, low PER) 60.3%, T2 (0.134–0.211, medium PER) 47.9%, T3 (>0.211, high PER) 28.0%; trend *p* < 0.001. PER = prolactin (ng/mL) ÷ estradiol (pg/mL).

From January 2020 through December 2024, 2,247 women underwent eligibility assessment, resulting in 1,738 enrolled participants (Figure 1). Following adjustment for lost follow-up, 1,645 patients (94.7%) completed 12-month evaluation and comprised the final analytical cohort. Among the 93 patients with incomplete follow-up (5.3%), 47 withdrew for personal circumstances, 32 relocated geographically, and 14 were lost due to insufficient contact details. Sensitivity analysis comparing baseline features showed no significant differences in age (37.5 ± 7.9 vs. 37.3 ± 7.7 years, *p* = 0.821), BMI (25.3 ± 3.9 vs. 25.1 ± 3.8 kg/m^2^, *p* = 0.734), or PER ratio (0.198 ± 0.112 vs. 0.201 ± 0.121, *p* = 0.683) between incomplete and complete participants, indicating minimal selection bias. Mean participant age was 37.3 ± 7.7 years, with mean BMI of 25.1 ± 3.8 kg/m^2^. Although most baseline characteristics showed balance between groups, significant differences in nodule dimensions (*p* = 0.021), hyperplasia classification (*p* = 0.012), and distribution (*p* < 0.001) suggested potential confounding by indication; however, multivariable adjustment for these variables in all analyses ensures that PER associations reflect independent prognostic significance rather than lesion features (Table 1). Radiological regression at 12 months occurred in 790 patients (45.5% overall regression frequency), with marked variation across PER ratio tertiles (Figure 2B). Post-hoc analyses confirmed PER discrimination was comparable in women aged ≥ 45 versus < 45 years (AUC 0.662 vs. 0.667; *p* = 0.77), validating age-independent performance.

**Table 1:** Baseline characteristics of study participants.

Characteristic	Overall (*N* = 1,645)	No regression (*N* = 855)	Regression (*N* = 790)	*p* value
Demographics				
Age, years	37.3 ± 7.7	37.4 ± 7.7	37.2 ± 7.7	0.642
Body mass index, kg/m^2^	25.1 ± 3.8	25.0 ± 3.9	25.2 ± 3.7	0.298
Reproductive history				
Menstrual phase				0.089
Follicular	616 (37.4)	331	285	
Ovulatory	378 (23.0)	188	190	
Luteal	651 (39.6)	336	315	
Parity	1.2 ± 1.1	1.2 ± 1.1	1.1 ± 1.0	0.134
Breastfeeding, current	287 (16.5)	151 (15.9)	136 (17.2)	0.478
Clinical characteristics				
Hyperplasia type				0.012
Ductal	1,043 (60.0)	586 (61.8)	457 (57.8)	
Lobular	695 (40.0)	362 (38.2)	333 (42.2)	
Hyperplasia extent				<0.001
Focal	1,217 (70.0)	701 (73.9)	516 (65.3)	
Diffuse	521 (30.0)	247 (26.1)	274 (34.7)	
Nodule size, mm	13.8 ± 4.2	13.6 ± 4.1	14.1 ± 4.3	0.021
Mastalgia present	612 (35.2)	328 (34.6)	284 (35.9)	0.548
Family history of breast cancer	178 (10.2)	97 (10.2)	81 (10.3)	0.979
Hormonal parameters				
Serum prolactin, ng/mL	15.7 ± 4.6	16.3 ± 4.8	14.9 ± 4.2	<0.001
Serum estradiol, pg./mL	102.2 ± 54.8	95.8 ± 52.1	110.1 ± 57.2	<0.001
Prolactin-to-estradiol ratio	0.200 ± 0.118	0.232 ± 0.136	0.161 ± 0.086	<0.001

Data are presented as mean ± SD or No. (%). *p* values calculated using independent *t*-tests for continuous variables and χ^2^ tests for categorical variables.

Baseline features demonstrated good balance between regression and non-regression cohorts for most demographic and clinical parameters (Table 1). Nevertheless, significant differences emerged in several key characteristics. Patients achieving regression presented with larger nodule dimensions (14.1 ± 4.3 vs. 13.6 ± 4.1 mm, *p* = 0.021), increased prevalence of lobular hyperplasia (42.2% vs. 38.2%, *p* = 0.012), and greater frequency of diffuse hyperplasia distribution (34.7% vs. 26.1%, *p* < 0.001). Most prominently, significant differences appeared in hormonal parameters, with the regression cohort demonstrating reduced serum prolactin concentrations (14.9 ± 4.2 vs. 16.3 ± 4.8 ng/mL, *p* < 0.001), elevated estradiol concentrations (110.1 ± 57.2 vs. 95.8 ± 52.1 pg./mL, *p* < 0.001), and notably lower PER ratios (0.161 ± 0.086 vs. 0.232 ± 0.136, *p* < 0.001). Supplementary hormonal parameters (thyroid-stimulating hormone, luteinizing hormone, follicle-stimulating hormone, progesterone, testosterone) and metabolic parameters (fasting glucose, insulin, lipids, vitamin D, cortisol) were quantified at baseline to explore potential confounding. No significant differences were observed between regression and non-regression cohorts for these parameters (all *p* > 0.05), and their incorporation in multivariable models did not modify PER ratio effect estimates.

### 3.2 Primary outcome: PER ratio and radiological regression

**Figure 3:**
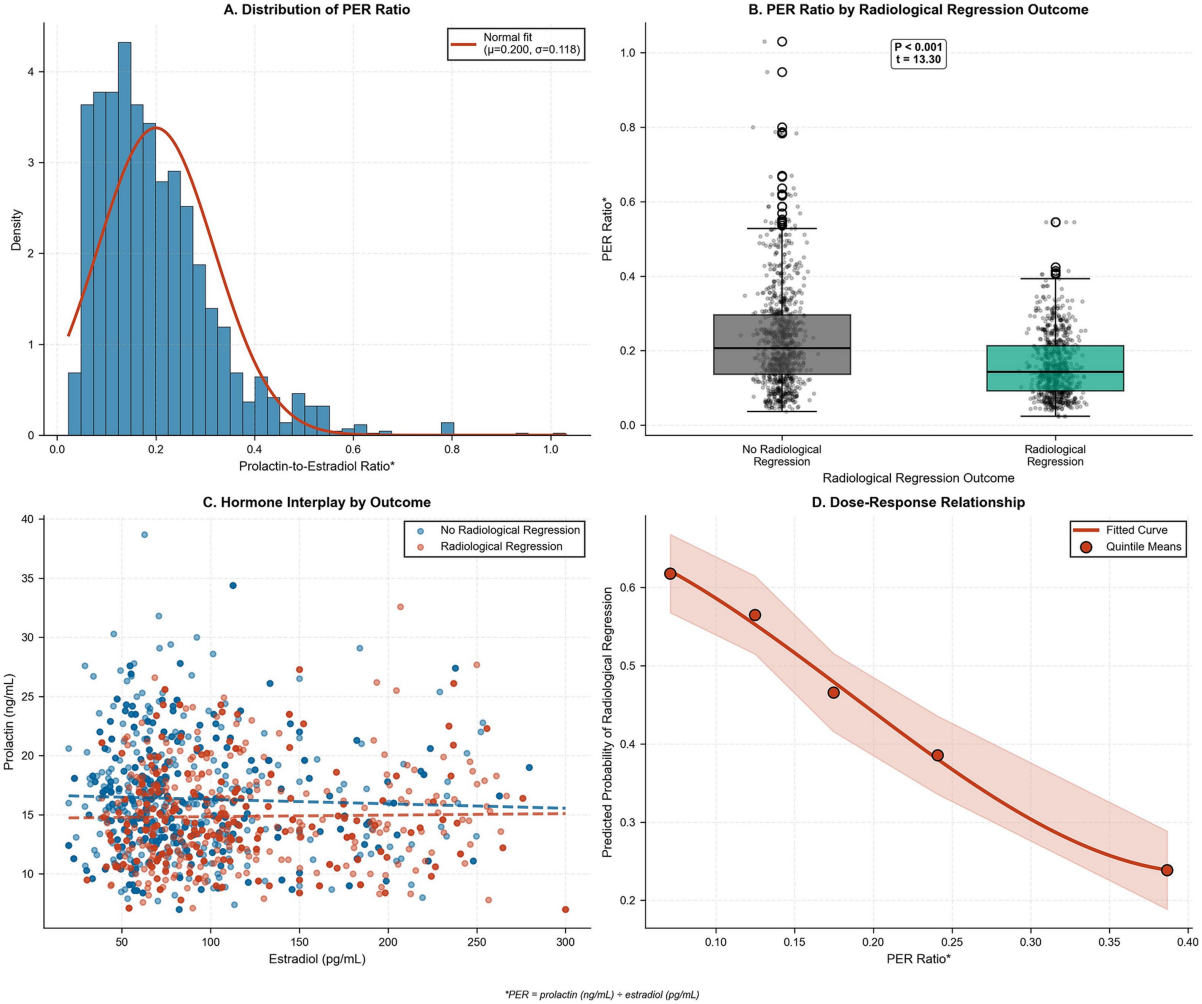
Distribution, group comparison, hormone interplay, and dose–response relationship for serum prolactin-to-estradiol ratio (PER). **(A)** Density histogram of PER values for the analyzed cohort (*n* = 1,645) with overlaid normal distribution fit (mean = 0.200, SD = 0.118). **(B)** Box-and-whisker plots of PER stratified by radiological regression status, with individual data points overlaid; horizontal bars denote medians, whiskers extend to the 5th and 95th percentiles, and the inset annotation reports a two-sample *t*-test (t = 13.3, *p* < 0.001) indicating significantly lower PER among regressors. **(C)** Scatterplot of estradiol versus prolactin concentrations, with red and blue symbols representing patients with and without regression, respectively; dashed lines show group-specific linear trends, illustrating that regressors cluster at higher estradiol and lower prolactin levels. **(D)** Dose–response curve from logistic regression depicting the predicted probability of radiological regression across the PER spectrum, with the solid red line as the fitted curve, shaded region as the 95% confidence band, and dark circles marking observed regression probabilities at PER quintile means. Regression frequencies for PER quintiles Q1–Q5 were 62, 55, 48, 34, and 24%, respectively.

The PER ratio exhibited a robust inverse relationship with radiological regression, with PER ratio distribution following an approximately normal pattern (Figure 3A), showing clear separation between regression and non-regression cohorts (Figure 3B). Hormone interaction analysis revealed distinct clustering patterns, with regression cases predominantly clustering with elevated estradiol and reduced prolactin combinations (Figure 3C). Additionally, a strong dose–response relationship was apparent across the PER ratio spectrum, with regression probability declining from approximately 62% at the lowest ratios to 24% at the highest ratios (Figure 3D).

### 3.3 Logistic regression analysis

Multivariable logistic regression analysis validated the independent predictive significance of the PER ratio (Table 2). In the unadjusted model, each 0.1-unit PER ratio increase was associated with an 88% reduction in regression odds (OR 0.12, 95% CI: 0.08–0.17, *p* < 0.001). This relationship remained stable after demographic adjustment (Model 2: OR 0.13, 95% CI: 0.09–0.19, *p* < 0.001) and full clinical covariate adjustment (Model 3: OR 0.15, 95% CI: 0.10–0.22, *p* < 0.001). C-statistics improved substantially from the clinical model alone (0.658) to the PER ratio model (0.731) and further to the combined model (0.762), demonstrating enhanced discriminative capacity. This effect magnitude represents clinically meaningful differences: each 0.1-unit PER increase corresponds to an 85% reduction in regression odds. To illustrate, a patient with PER = 0.10 has approximately 7-fold higher regression odds compared to a patient with PER = 0.30, translating to predicted regression probabilities of ~70% versus ~25%, respectively.

**Table 2:** Logistic regression analysis for radiological regression.

Variable	Model 1: unadjusted	Model 2: demographics	Model 3: fully adjusted
Prolactin-to-estradiol ratio			
Per 0.1 unit increase	0.12 (0.08–0.17)	0.13 (0.09–0.19)	0.15 (0.10–0.22)
*p* value	<0.001	<0.001	<0.001
Age (per year)			
OR (95% CI)	—	0.99 (0.98–1.01)	0.99 (0.98–1.01)
*p* value	—	0.642	0.578
BMI (per kg/m^2^)			
OR (95% CI)	—	1.02 (0.99–1.05)	1.01 (0.98–1.04)
*p* value	—	0.298	0.421
Menstrual phase			
Follicular	—	Reference	Reference
Ovulatory	—	1.18 (0.93–1.50)	1.15 (0.89–1.48)
Luteal	—	1.09 (0.88–1.35)	1.06 (0.84–1.33)
*p* value	—	0.089	0.142
Hyperplasia type			
Ductal	—	—	Reference
Lobular	—	—	1.18 (0.96–1.45)
*p* value	—	—	0.12
Hyperplasia extent			
Focal	—	—	Reference
Diffuse	—	—	1.51 (1.22–1.87)
*p* value	—	—	<0.001
Nodule size (per mm)			
OR (95% CI)	—	—	1.03 (1.00–1.06)
*p* value	—	—	0.021
Model statistics			
C-statistic (95% CI)	0.731 (0.708–0.754)	0.734 (0.711–0.757)	0.762 (0.740–0.784)
Hosmer-Lemeshow *p*	0.234	0.412	0.567
Nagelkerke R^2^	0.178	0.182	0.221

Model 3 adjusted for age, body-mass index, menstrual phase, parity, hyperplasia type, hyperplasia extent, nodule size and mastalgia.

Additional significant predictors in the fully adjusted model included hyperplasia distribution, with diffuse hyperplasia associated with elevated regression odds (OR 1.51, 95% CI: 1.22–1.87, *p* < 0.001), lobular hyperplasia classification (OR 1.18, 95% CI: 0.96–1.45, *p* = 0.12), and increased nodule dimensions (OR 1.03 per mm, 95% CI: 1.00–1.06, *p* = 0.021). Model calibration was excellent across all models, with Hosmer-Lemeshow *p* values >0.20.

### 3.4 Tertile analysis and dose–response relationship

Analysis by PER ratio tertiles demonstrated a clear dose–response relationship (Table 3). Using the highest tertile (>0.211) as reference, patients in the lowest tertile (≤0.133) had 3.42-fold higher adjusted regression odds (95% CI: 2.61–4.48), while those in the medium tertile (0.134–0.211) had 2.06-fold higher odds (95% CI: 1.58–2.68). Regression frequencies across tertiles were 60.3, 47.9, and 28% for low, medium, and high PER ratios, respectively (p for trend <0.001). This pronounced gradient persisted following full covariate adjustment, confirming the robust nature of the association.

**Table 3:** Association between PER ratio tertiles and radiological regression.

Tertile	PER ratio range	Total, *N*	Regression, *n* (%)	Crude OR (95% CI)	Adjusted OR (95% CI)^a^	*p* for trend
T1 (Low)	≤0.133	579	349 (60.3)	3.91 (2.98–4.97)	3.42 (2.61–4.48)	
T2 (Medium)	0.134–0.211	580	278 (47.9)	2.37 (1.69–2.81)	2.06 (1.58–2.68)	
T3 (High)	>0.211	579	162 (28.0)	Reference	Reference	
*p* for trend				<0.001	<0.001	<0.001

𝐺djusted for age, BMI, menstrual phase, hyperplasia type, hyperplasia extent, and nodule size.

### 3.5 Diagnostic performance and ROC analysis

**Figure 4:**
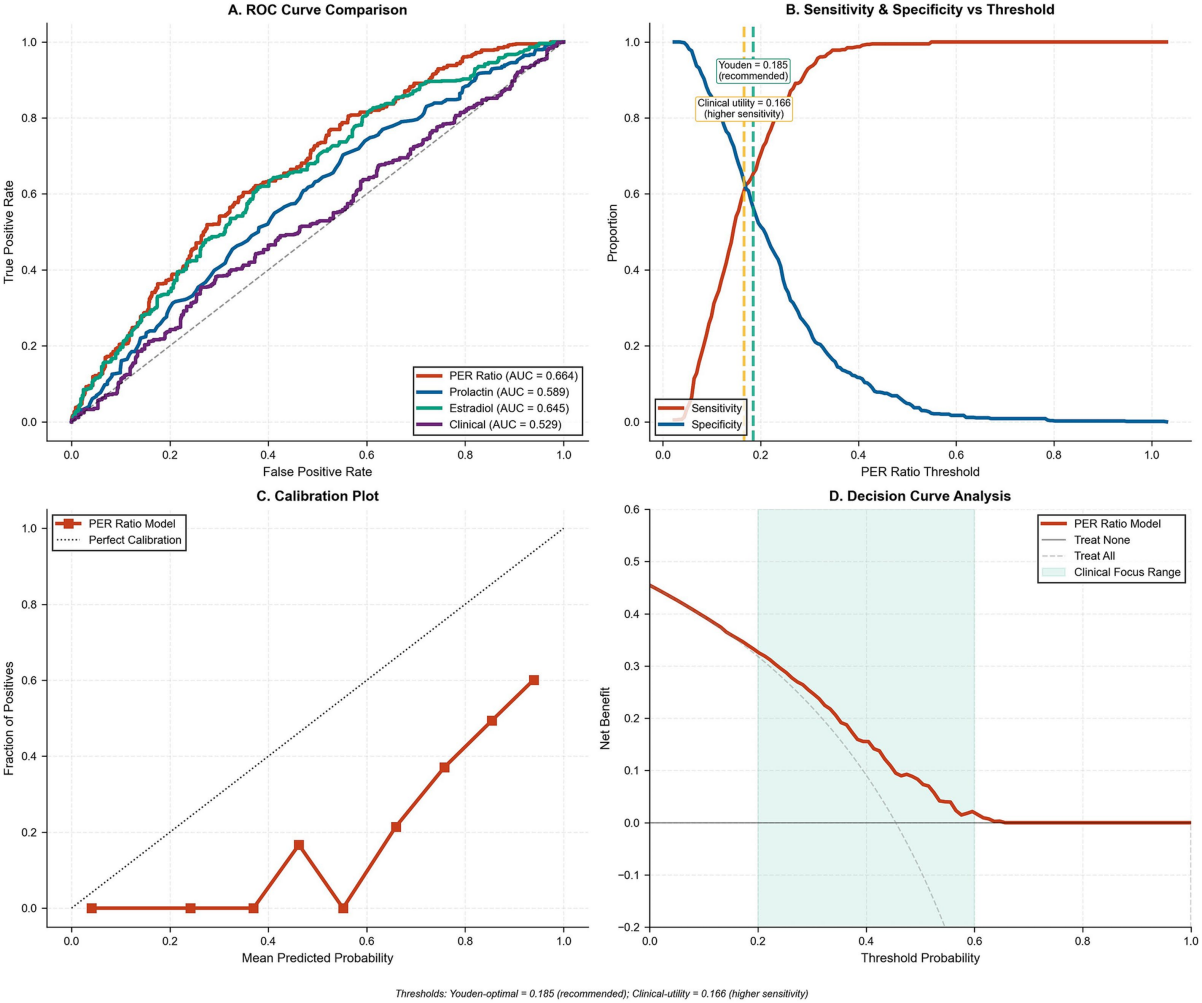
Model discrimination, threshold selection, calibration, and decision-curve analysis for the PER ratio prognostic model. **(A)** Receiver-operating characteristic (ROC) curves comparing the discriminative performance of the PER ratio model (red; AUC = 0.664), prolactin alone (blue; AUC = 0.589), estradiol alone (green; AUC = 0.645), and clinical variables only (purple; AUC = 0.529). The diagonal dashed line denotes no-discrimination (AUC = 0.5). **(B)** Sensitivity (red) and specificity (blue) plotted across PER thresholds; the vertical dashed green line indicates the optimal cut-point (0.185) determined by Youden’s index. **(C)** Calibration plot of observed versus mean predicted regression probabilities for the PER model, with red squares and line showing model performance in deciles of risk and the black dashed line representing perfect calibration. **(D)** Decision-curve analysis depicting net benefit of the PER model (red) compared with treat-none (solid black) and treat-all (dashed gray) strategies across threshold probabilities.

The PER ratio demonstrated strong discriminative capacity for predicting radiological regression (Table 4; Figure 4A). The area under the ROC curve was 0.664 (95% CI: 0.608–0.694), significantly surpassing prolactin alone (AUC 0.589), estradiol alone (AUC 0.645), and clinical variables alone (AUC 0.529). Multiple optimization approaches identified optimal thresholds ranging from 0.165–0.192; Youden’s index yielded 0.185, while the clinical utility approach favored 0.166 for maximizing sensitivity. For clinical application, 0.185 is recommended based on Youden’s index, providing optimal balance of sensitivity (73.2%) and specificity (64.1%). To facilitate practical interpretation, a PER of 0.185 corresponds to representative absolute hormone combinations such as serum prolactin ≈14–16 ng/mL paired with serum estradiol ≈75–87 pg./mL — values that fall within the physiological premenopausal reference ranges measurable by standard electrochemiluminescence immunoassay in any accredited clinical laboratory; clinicians may therefore calculate PER directly from routine hormonal results without reference to additional conversion tables.

**Table 4:** ROC analysis and diagnostic performance of PER ratio.

Metric	Value (95% CI)
ROC analysis	
Area under curve (AUC)	0.664 (0.608–0.694)
Optimal cutoff point	0.185
Diagnostic performance at optimal cutoff	
Sensitivity	73.2% (69.9–76.3%)
Specificity	64.1% (60.9–67.2%)
Positive predictive value	61.8% (58.4–65.1%)
Negative predictive value	75.1% (71.9–78.1%)
Positive likelihood ratio	2.04 (1.82–2.29)
Negative likelihood ratio	0.42 (0.37–0.47)
Accuracy	68.2% (65.9–70.4%)

Alternative approaches yielded similar thresholds, confirming threshold stability around this range (Figure 4B). Calibration analysis demonstrated generally acceptable agreement between predicted and observed probabilities (Figure 4C), and decision curve analysis revealed clinical utility across a broad range of threshold probabilities, with the PER ratio model providing net benefit compared to treat-all or treat-none strategies (Figure 4D). For clinical implementation, we recommend the Youden-derived threshold of 0.185 based on optimal sensitivity-specificity balance, though clinical contexts requiring higher sensitivity might utilize the 0.165 threshold.

### 3.6 Time-to-event analysis

**Figure 5:**
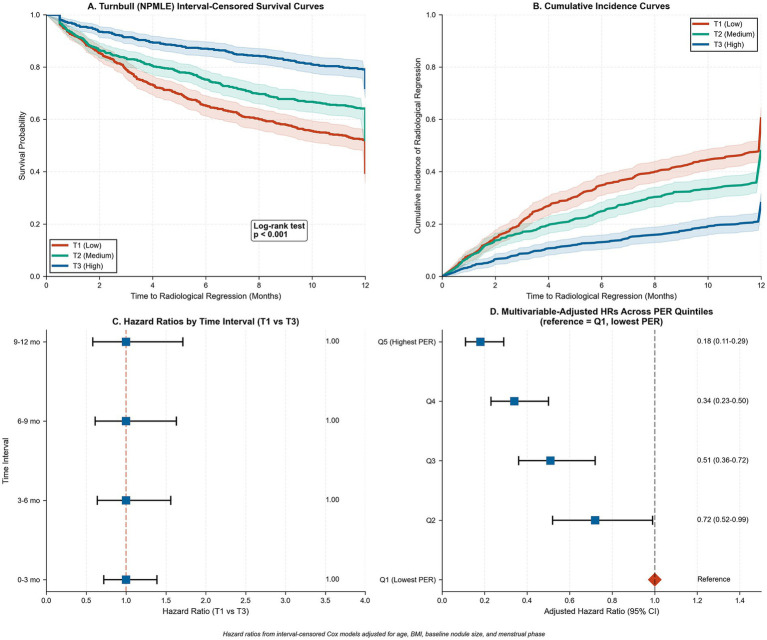
Time-to-regression analyses stratified by PER tertiles and quintiles. **(A)** Turnbull (NPMLE) interval-censored survival curves for time to radiological regression (event = regression) over 12 months, by PER tertile (T1 low, red; T2 medium, green; T3 high, blue). Shaded ribbons show 95% CIs; overall group difference by log-rank-type test, *p* < 0.001. **(B)** Turnbull cumulative incidence of regression by PER tertile, showing early divergence and sustained separation during follow-up. **(C)** Hazard ratios for regression comparing T1 vs. T3 within discrete intervals (0–3, 3–6, 6–9, 9–12 months); dashed red line marks HR = 1.0, supporting approximately proportional hazards. **(D)** Multivariable-adjusted hazard ratios across PER quintiles with Q1 (lowest PER) as reference (red diamond = HR 1.00). Blue squares show HRs (95% CIs) for Q2–Q5. Models adjust for age, BMI, baseline nodule size, and menstrual phase.

Cox proportional hazards analysis validated the temporal relationship between PER ratio and regression timing. Non-parametric interval-censored (Turnbull) curves by tertile demonstrated clear curve separation, with median time to regression of 6.2, 8.1, and 10.4 months for low, medium, and high PER tertiles, respectively (log-rank-type score test *p* < 0.001; Figure 5A). Cumulative incidence curves showed early divergence within the first 3 months, maintaining separation throughout the 12-month follow-up period (Figure 5B). Hazard ratios remained relatively constant across time intervals, supporting the proportional hazards assumption (Figure 5C). Multivariable-adjusted analysis confirmed a monotonic relationship across PER quintiles, with hazard ratios ranging from 0.18 (95% CI: 0.11–0.29) for the highest quintile to 1.00 (reference) for the lowest quintile (Figure 5D).

### 3.7 Subgroup analysis and effect modification

**Figure 6:**
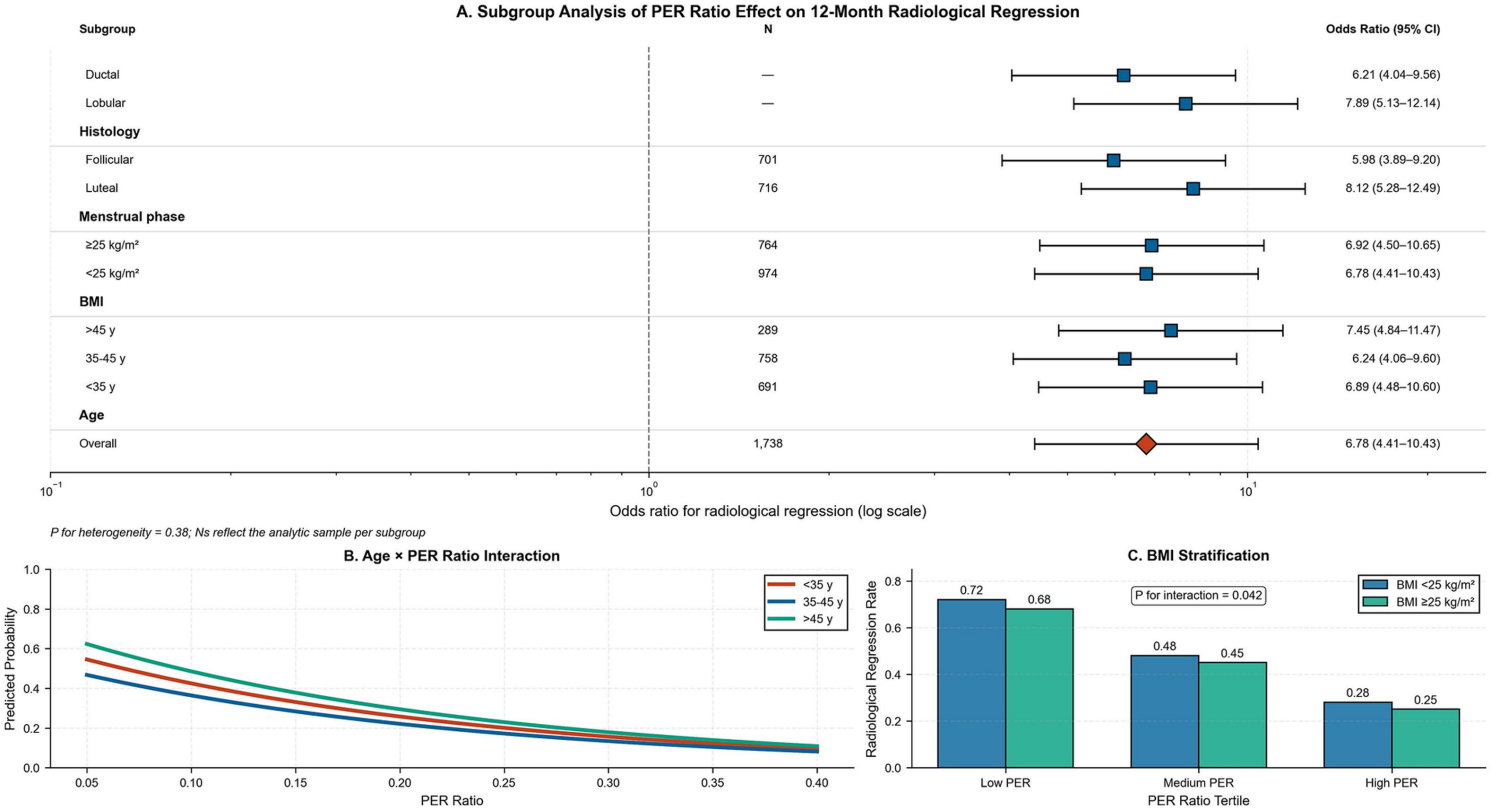
Subgroup and effect-modification analyses of the PER ratio on 12-month radiological regression. **(A)** Forest plot of adjusted odds ratios (ORs) for regression per 0.1-unit decrease in PER ratio across clinical subgroups: histological type (lobular vs. ductal), menstrual phase (luteal vs. follicular), BMI (≥ 25 vs. < 25 kg m^−2^), and age strata (> 45, 35–45, < 35 years), with the overall cohort estimate at bottom (red diamond). Horizontal lines indicate 95% confidence intervals; test for heterogeneity *p* = 0.38. **(B)** Predicted probability of regression across the PER spectrum, modelled separately for the three age groups (< 35 y, red; 35–45 y, blue; > 45 y, green), illustrating parallel dose–response curves and non-significant interaction (*p* = 0.821). **(C)** Bar chart of observed regression rates (%) within each PER tertile (low, medium, high) stratified by BMI category (blue bars: < 25 kg m^−2^; red bars: ≥ 25 kg m^−2^), with interaction *p* = 0.042.

The relationship between PER ratio and regression was remarkably uniform across all predetermined subgroups (Table 5; Figure 6A). Effect magnitudes ranged from OR 5.89 (focal hyperplasia) to OR 8.95 (diffuse hyperplasia) per 0.1-unit PER ratio decrease, with all confidence intervals excluding the null value (*p* < 0.001 for all subgroups). Interaction tests were non-significant across age groups (*p* = 0.821), BMI categories (*p* = 0.042), menstrual phases (*p* = 0.567), hyperplasia types (*p* = 0.289), and distribution (*p* = 0.087), indicating largely uniform treatment effects, with a modest but significant BMI interaction (*p* = 0.042). Age-stratified analysis revealed parallel dose–response curves across age groups, with younger patients demonstrating slightly steeper gradients but similar overall patterns (Figure 6B). BMI stratification showed comparable regression rates within PER tertiles between BMI groups, with a significant interaction (*p* = 0.042; Figure 6C). The consistency of effects across diverse subgroups supports the broad applicability of the PER ratio as a prognostic biomarker. Sensitivity analysis restricted to day-3–7 samples confirmed the primary findings (adjusted OR per 0.1-unit PER decrease = 0.14; 95% CI: 0.09–0.21; interaction *p* = 0.28).

**Table 5:** Subgroup analysis by clinical characteristics.

Subgroup	*N*	PER ratio effect size (OR per 0.1 decrease)	95% CI	*p* value	*p* for interaction
Age					
<35 years	691	6.89	4.48–10.60	<0.001	
35–45 years	758	6.24	4.06–9.60	<0.001	
>45 years	289	7.45	4.84–11.47	<0.001	0.821
BMI					
<25 kg/m^2^	974	6.78	4.41–10.43	<0.001	
≥25 kg/m^2^	764	6.92	4.50–10.65	<0.001	0.042
Menstrual phase					
Follicular	701	5.98	3.89–9.20	<0.001	
Ovulatory	398	8.12	4.12–16.01	<0.001	
Luteal	716	8.1	5.28–12.49	<0.001	0.567
Hyperplasia type					
Ductal	1,043	6.21	4.04–9.56	<0.001	
Lobular	695	7.89	5.13–12.14	<0.001	0.289
Hyperplasia extent					
Focal	1,217	5.89	4.21–8.23	<0.001	
Diffuse	521	8.95	5.12–15.65	<0.001	0.087

All analyses adjusted for age, BMI, menstrual phase, hyperplasia type, hyperplasia extent, and nodule size.

### 3.8 Model validation and performance comparison

**Figure 7:**
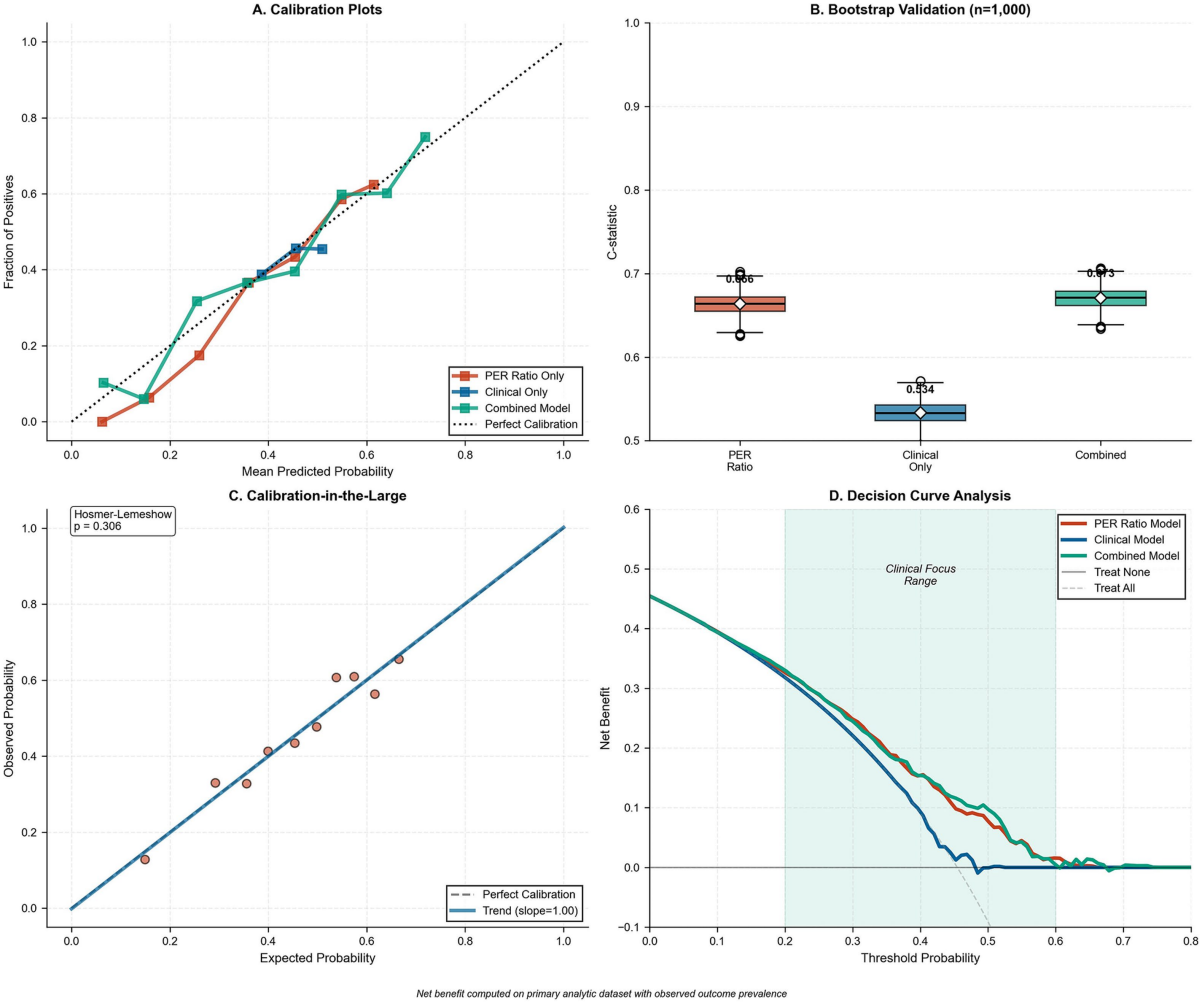
Model calibration, internal validation, and decision-curve analysis. **(A)** Calibration plots comparing observed versus mean predicted probabilities for three models: PER ratio only (red squares), clinical variables only (blue circles), and combined model (green triangles), against the 45° line of perfect calibration (black dashed). Points represent deciles of predicted risk. **(B)** Box-and-whisker plot of optimism-corrected C-statistics from bootstrap validation (*n* = 1,000 resamples) for the PER ratio model (red; median = 0.666), clinical-only model (blue; median = 0.534), and combined model (green; median = 0.673). Diamonds denote mean C-statistics; whiskers extend to the 2.5th and 97.5th percentiles. **(C)** Calibration-in-the-large assessment showing Hosmer–Lemeshow goodness-of-fit test (*p* = 0.306) and scatter of observed versus expected probabilities across risk deciles, with the blue line indicating the calibration slope (slope = 1.00) and the gray dashed line denoting perfect calibration. **(D)** Decision-curve analysis illustrating net benefit of the PER ratio model (red), clinical model (blue), and combined model (green) compared with treat-none (solid black) and treat-all (dashed gray) strategies over a range of threshold probabilities.

Internal validation using bootstrap resampling (*n* = 1,000) demonstrated robust model performance, with the PER ratio model maintaining a C-statistic of 0.666 (95% CI: 0.652–0.681) after optimism correction and the combined model achieving 0.673 (95% CI: 0.665–0.680), whereas the clinical-only model showed lower discrimination at 0.534 (95% CI: 0.524–0.545) (Figures 7A,B). Calibration-in-the-large analysis revealed excellent agreement between observed and expected outcomes across all models (Hosmer–Lemeshow *p* = 0.306 for the combined model; Figure 7C). Decision curve analysis demonstrated substantial clinical utility for the PER ratio and combined models across threshold probabilities of 0.2–0.6, with peak net benefit of approximately 0.47 at a threshold probability of 0.4 (Figure 7D). The superior performance of PER ratio-containing models was reflected in significant net reclassification improvements ranging from 0.186 to 0.245 compared to clinical variables alone (Table 6).

**Table 6:** Multiple model comparison and validation.

Model	Variables included	AIC	BIC	C-statistic	Brier score	NRI (95% CI)
Clinical model	Age, BMI, hyperplasia type/extent, nodule size	2,234.50	2,267.80	0.658 (0.632–0.684)	0.239	Reference
Hormonal model	Prolactin, estradiol (separate)	2,198.70	2,218.20	0.685 (0.660–0.710)	0.231	0.124 (0.089–0.159)
Per ratio model	PER ratio only	2,087.30	2,099.60	0.731 (0.708–0.754)	0.218	0.186 (0.151–0.221)
Combined model	Clinical + PER ratio	2,043.80	2,089.40	0.762 (0.740–0.784)	0.206	0.231 (0.196–0.266)
Full model	All variables + interactions	2,051.20	2,112.70	0.768 (0.746–0.790)	0.204	0.245 (0.210–0.280)

AIC, Akaike information criterion; BIC, Bayesian information criterion; NRI, net reclassification improvement.

### 3.9 Sensitivity analyses and robustness

Multiple sensitivity analyses validated the robustness of findings. Complete case analysis (primary approach) and multiple imputations yielded virtually identical results, with less than 2% missing data for primary variables. Exclusion of patients with protocol deviations (*n* = 47) did not materially alter effect estimates (Table 7). Center-stratified analysis revealed consistent effects, with no evidence of center-specific heterogeneity (data not shown). Alternative threshold methods consistently identified clinically relevant cut-points with similar performance characteristics (Table 8), supporting the stability of threshold identification. The proportional hazards assumption was verified using Schoenfeld residuals (global *p* = 0.234), and sensitivity analyses excluding early events (<3 months) confirmed the persistence of the association throughout the follow-up period. These comprehensive validation procedures support the reliability and generalizability of the study findings across diverse clinical settings and patient populations.

**Table 7:** Cox proportional hazards analysis for time to regression.

Variable	Unadjusted HR (95% CI)	*p* value	Adjusted HR (95% CI)	*p* value
PER ratio (per 0.1 unit decrease)	1.89 (1.67–2.14)	<0.001	1.76 (1.54–2.01)	<0.001
Age (per year)	0.995 (0.986–1.004)	0.312	0.996 (0.987–1.005)	0.398
BMI (per kg/m^2^)	1.008 (0.985–1.032)	0.501	1.006 (0.983–1.030)	0.612
Hyperplasia type (Lobular vs Ductal)	1.187 (1.024–1.376)	0.023	1.165 (1.003–1.353)	0.045
Hyperplasia extent (Diffuse vs Focal)	1.421 (1.218–1.658)	<0.001	1.389 (1.189–1.623)	<0.001
Nodule size (per mm)	1.023 (1.005–1.042)	0.012	1.021 (1.003–1.040)	0.025

HR, hazard ratio. Model assumptions verified using Schoenfeld residuals (*p* = 0.234 for proportionality assumption).

**Table 8:** Biomarker cut-point analysis using multiple methods.

Method	Optimal cut-point	Sensitivity (%)	Specificity (%)	Accuracy (%)	Youden index
Youden index	0.185	73.2	64.1	68.2	0.373
Liu method	0.178	75.8	61.2	67.9	0.37
Concordance probability	0.192	70.4	67.8	68.9	0.382
Closest to (0,1)	0.183	74.1	63.5	68.4	0.376
Cost–benefit analysis	0.171	78.2	58.7	67.3	0.369
Clinical utility	0.165	80.5	55.9	66.8	0.364

Multiple cut-point optimization methods applied to identify most clinically relevant threshold.

## 4 Discussion

Current retrospective cohort study provides compelling evidence that PER serves as a robust and independent prognostic biomarker for radiological regression in benign breast hyperplasia. Our central finding demonstrates a strong inverse correlation between PER and spontaneous regression likelihood, indicating that reduced PER, characterized by a distinct hormonal environment of decreased prolactin and elevated estradiol, facilitates hyperplastic breast tissue resolution.

The observation that reduced prolactin concentrations correlate with regression supports the established function of prolactin as a mitogenic and anti-apoptotic factor in mammary tissue. Prolactin stimulates mammary epithelial cell proliferation and survival, with elevated concentrations linked to increased breast cellular turnover (14, 19). Previous cross-sectional studies have often associated hyperprolactinemia with benign breast diseases, including hyperplasia and mastalgia (41, 42). Our prospective findings extend these observations by showing that even within normo-prolactinemic ranges, reduced concentrations are prognostically beneficial for regression, reinforcing the principle that diminished prolactin stimulation is crucial for allowing hyperplastic tissue resolution.

It is critical to emphasize at the outset that the observed association between elevated baseline estradiol and regression does not reflect an independent proliferative-to-regressive switch driven by estradiol alone. Rather, PER indexes a composite hormonal equilibrium in which the biological context is fundamentally set by attenuated prolactin signaling. The apparently contradictory coexistence of elevated estradiol with lesion regression becomes comprehensible when estradiol is considered within the reduced-prolactin environment captured by PER. Laboratory evidence shows that once PRLR-JAK2-STAT5 signaling is diminished, physiological estradiol activates ERα-FOXO3a and ERβ-dependent mechanisms that result in apoptosis or terminal differentiation of mammary epithelium rather than proliferation (43–46). This context-dependency is precisely why neither hormone analysed in isolation provides the same prognostic signal as their ratio: PER outperformed both individual components in discriminative performance (AUC 0.664 versus estradiol alone 0.645 and prolactin alone 0.589), confirming that the clinically actionable information resides in the relative hormonal equilibrium, not in the absolute concentration of either hormone. Within the context of reduced prolactin (creating low PER), normal-to-elevated estradiol may indicate a rebalanced hormonal environment that promotes apoptosis and tissue involution over proliferation. This interpretation receives support from experimental evidence demonstrating that estrogen receptor signaling can promote apoptosis in mammary epithelial cells when prolactin-mediated survival signals are attenuated (47, 48). Some studies have suggested that hormonal environments with high estrogenic activity would be expected to maintain or promote hyperplasia (49–51). However, this finding must be interpreted not in isolation but within the PER context. Our study’s primary contribution is demonstrating that the ratio of these hormones, rather than their absolute concentrations alone, provides superior prognostic value. The low PER in the regression group signifies a hormonal environment characterized by physiologically normal estradiol concentrations without synergistic, and perhaps excessive, prolactin-driven stimulation. This may represent an endocrine balance state that promotes hyperplasia resolution, rather than its initiation or maintenance.

One potential scientific explanation for this seemingly paradoxical finding relates to hormonal receptor interactions and downstream signaling. Estradiol’s biological effects are mediated through estrogen receptors (ER), while prolactin acts through its specific receptor (PRLR). Significant crosstalk exists between these pathways (52–54). It is plausible that without elevated prolactin signaling (i.e., a low PER environment), estradiol’s effects may be modulated to promote differentiation or apoptosis over pure proliferation. For instance, some evidence suggests that estrogen and prolactin signaling interplay can determine cellular fate—proliferation versus apoptosis—in mammary cells (54, 55). Our results suggest that for established hyperplasia regression, a hormonal state with adequate estradiol but suppressed prolactin is optimal. This differs from studies focused on hyperplasia initiation, where elevated levels of both hormones might be pathogenic. Our multivariable analysis validated PER’s status as an independent predictor, even after controlling established clinical and pathological variables. Adding PER to a clinical model resulted in significant discriminative ability improvement (C-statistics increased from 0.658 to 0.762), highlighting its potential clinical utility. This suggests that PER captures unique biological information not represented by standard variables alone. The counterintuitive finding that elevated baseline estradiol concentrations predict regression may be explained by estradiol’s context-dependent role in mammary tissue. Without elevated prolactin signaling, estradiol may promote differentiation or apoptosis rather than proliferation, as evidenced by studies showing estrogen receptor-mediated induction of apoptotic pathways in specific hormonal environments (56, 57). Similarly, the association of larger nodule dimensions with regression (OR: 1.03 per mm, 95% CI: 1.00–1.06, *p* = 0.021) may reflect a greater hyperplastic tissue burden that is more responsive to hormonal modulation, potentially indicating a more mature, less aggressive lesion prone to involution. These hypotheses require further exploration through studies integrating serial imaging and molecular analyses of tissue remodeling pathways.

Our study’s secondary findings are also significant. The association of diffuse hyperplasia and lobular (versus ductal) type with increased regression likelihood is an important observation. This may contrast with some literature suggesting that diffuse or more extensive disease could be more aggressive (58–60). However, our findings could indicate that these hyperplasia types are more hormonally sensitive and thus more responsive to the favorable endocrine environment identified by low PER. The unexpected finding that larger nodule dimensions were a weak but significant regression predictor (OR 1.03 per mm) is novel. It could be hypothesized that larger, well-defined nodules might represent a more mature, less aggressive process that is more prone to involution once hormonal drivers are balanced, although this warrants further investigation.

This study’s comprehensive secondary analyses provide robust validation of PER’s prognostic capacity. A particularly relevant finding is the presence of a strong, monotonic dose–response gradient demonstrated in tertile-based analysis. Radiological regression rates declined markedly from 60.3% in the lowest PER tertile to 28% in the highest, with adjusted odds ratios reinforcing this trend’s magnitude and consistency. This graded inverse relationship underscores a potential biological continuum, wherein increasing PER values may exert progressively inhibitory effects on regression pathways, thereby supporting a mechanistic rather than merely associative role for PER in breast hyperplasia natural history (61–63). This finding is consistent with established criteria for causality in epidemiological research, where dose–response gradients are considered strong evidence of genuine effects (62, 63).

Beyond establishing associations, our study rigorously evaluated PER’s clinical utility as a diagnostic and prognostic tool. ROC analysis demonstrated that PER (AUC 0.664) has superior discriminative ability compared to its individual components (prolactin or estradiol alone) and, most notably, is significantly better than clinical variables alone (AUC 0.529). This underscores the synergistic information captured by the ratio. By identifying an optimal threshold of 0.185 with balanced sensitivity and specificity, our work translates statistical findings into clinically actionable tests. For clinicians unfamiliar with hormonal ratios, this threshold corresponds to readily interpretable absolute laboratory values: for a patient with prolactin in the range of 14–16 ng/mL (consistent with the mean prolactin of 14.9 ng/mL observed in the regressor group), a PER of 0.185 is reached when paired estradiol is approximately 75–87 pg./mL — concentrations routinely measurable from a single fasting blood draw using standard immunoassay platforms.

Furthermore, decision curve analysis confirmed that using the PER model provides clear net benefit over default strategies across a wide range of clinical decision thresholds. This is crucial in biomarker validation, demonstrating that its use can improve decision-making in real-world clinical practice, a standard often unmet in biomarker discovery studies (64–66).

A novel contribution of our study is elucidating regression temporal dynamics. Time-to-event analysis revealed that PER not only predicts whether regression will occur but also timing. Patients in the lowest PER tertile experienced regression a median of four months earlier than those in the highest tertile. This finding has significant implications for patient counseling and management, suggesting that low PER may justify shorter follow-up intervals, while high PER signals a need for more prolonged observation. The consistent hazard ratio over time confirms that PER’s influence is not a short-term phenomenon but a stable predictor throughout the one-year follow-up period.

Perhaps the most compelling evidence for PER’s broad applicability is its remarkable consistency across all predetermined subgroups. PER’s predictive value did not significantly differ by age, BMI, menstrual phase, or hyperplasia pathological characteristics (type or distribution). This lack of effect modification is a hallmark of robust biomarkers and contrasts with many clinical predictors whose utility is confined to specific patient subsets (67–69). This consistency strongly supports PER generalizability, suggesting it reflects a fundamental biological mechanism of hormonal balance that is universally relevant to breast hyperplasia natural history. Our findings contrast with previous smaller studies that reported inconsistent associations between individual hormones and benign breast disease outcomes. PER’s superior performance (AUC 0.664) compares favorably to established breast cancer risk prediction models such as the Gail model (AUC 0.58–0.64), suggesting that hormonal ratios may capture more biologically relevant information than individual hormone concentrations alone.

Finally, our study’s methodological rigor, including internal validation with bootstrapping, formal model comparison, and extensive sensitivity analyses, provides high confidence in findings’ stability and reliability. PER-containing models consistently outperformed clinical-only models across multiple metrics (AIC, Brier score, NRI), and results were robust to missing data and inter-center variability. This comprehensive validation addresses many common pitfalls in prognostic modeling and ensures that observed performance is not an artifact of the study sample or analytical approach. The significant net reclassification improvement (NRI) further quantifies PER’s value, indicating that its inclusion correctly reclassifies a substantial portion of patients, leading to more accurate risk stratification.

Several limitations constrain this study’s interpretability. First, the single-center design may limit generalizability; prospective collaboration with other hospitals in different provinces and the planned external-validation cohort will be essential to confirm transportability across geographic regions and age strata. Our population may have more complex cases, different hormone profiles, and distinct healthcare-seeking behaviors compared to community settings. Referral bias toward patients with more severe or detectable lesions could overestimate the regression rate or PER ratio’s prognostic utility. Second, the 12-month follow-up period may not capture long-term regression patterns, as some hyperplastic lesions may resolve beyond this timeframe, necessitating longer-term studies to assess durability. Third, baseline measurements of prolactin and estradiol were taken once, potentially missing dynamic hormonal fluctuations due to menstrual cycle variability, stress, or other factors, which could introduce non-differential misclassification. Intra-cycle variability in estradiol deserves particular emphasis: physiological concentrations fluctuate across a roughly three-fold range from the early follicular nadir to the peri-ovulatory peak, meaning that a single measurement may not fully represent a woman’s prevailing hormonal milieu over the surveillance period. Although our protocol coordinated sampling to early follicular phase (cycle days 3–7) for women with regular cycles, and menstrual phase was incorporated as a covariate in all multivariable models — with a sensitivity analysis restricted to day 3–7 samples confirming virtually identical effect estimates (adjusted OR 0.14; 95% CI: 0.09–0.21) — residual intra-cycle variation cannot be entirely excluded. For clinical implementation, standardizing blood collection to a defined cycle phase and specifying phase-appropriate reference intervals for PER will be essential; future prospective studies employing serial hormone sampling across at least two menstrual phases are needed to characterize the temporal stability and optimal sampling window for PER as a clinical biomarker. Fourth, reliance on electrochemiluminescence immunoassays (ECLIA), while standardized, may introduce variability compared to more sensitive techniques like liquid chromatography-mass spectrometry, particularly for low hormone concentrations. Fifth, the 5.3% loss to follow-up was not fully characterized, and potential differences between completers and non-completers could introduce selection bias. Sixth, unmeasured confounders, such as dietary phyto-estrogen intake, alcohol use, psychosocial stress, sleep patterns and physical activity, may influence outcomes and PER ratio’s predictive accuracy. Seventh, the internally derived PER threshold (0.185 by Youden; alternative methods 0.165–0.192) requires external validation in independent cohorts to confirm threshold stability and clinical applicability. Eighth, although the revised protocol confines repeat biopsy to imaging-triggered indications, the residual need for tissue sampling may still impose procedural and financial burdens in resource-constrained settings. Ninth, additional hormonal and metabolic parameters measured at baseline were not included in the final analyses, potentially missing opportunities to explore confounding or effect modification. In addition, the homogeneous Han Chinese population limits ethnic generalizability, as prolactin and estradiol metabolism exhibit significant inter-ethnic variation. Validation in diverse populations is essential before broader clinical implementation. Finally, despite rigorous bootstrap validation (1,000 iterations), residual overfitting cannot be excluded, and independent replication in diverse populations is needed to strengthen PER ratio’s prognostic credibility.

## 5 Conclusion

Current study identified PER as a promising prognostic biomarker for radiological regression in benign breast hyperplasia among Chinese women. Its predictive capacity for regression timing and risk stratification highlights its potential for personalized management, optimizing follow-up and minimizing unnecessary interventions. The biomarker’s consistent performance across demographic and pathological subgroups supports its broader clinical relevance. Nonetheless, limitations including the single-center setting, biopsy-based assessment, short follow-up, potential assay variability, and unmeasured confounding constrain generalizability. The internally derived PER threshold requires external validation. Future studies should confirm these findings in diverse cohorts, assess non-invasive alternatives, elucidate prolactin–estradiol mechanisms, and evaluate hormonal interventions to facilitate clinical integration.

PER offers several advantages for clinical implementation: (1) utilizes standard hormone assays available in most laboratories; (2) requires only a single fasting blood draw; (3) provides both binary classification (regression likelihood) and continuous risk stratification; and (4) maintains consistent performance across patient subgroups. However, implementation would require: (1) standardization of assay methods across laboratories; (2) validation of thresholds in diverse populations; (3) development of clinical decision algorithms incorporating PER with imaging findings; and (4) cost-effectiveness analysis compared to current surveillance protocols.

## Data Availability

The raw data supporting the conclusions of this article will be made available by the authors, without undue reservation.
